# Efficacy of Esketamine as an Adjuvant for Fascia Iliaca Compartment Block on Postoperative Analgesia in Elderly Patients Undergoing Total Hip Arthroplasty

**DOI:** 10.12669/pjms.42.9.15889

**Published:** 2026-09

**Authors:** Xuandong Jia, Lidong Zhang, Maitao Zhou

**Affiliations:** 1 Xuandong Jia, Jinling Clinical Medical College, Nanjing Medical University, Nanjing, Jiangsu 210000, China., Department of Anesthesiology, The 904th Hospital of the Joint Logistic Support Force of PLA, Wuxi, Jiangsu 214000, China); 2 Lidong Zhang, Jinling Clinical Medical College, Nanjing Medical University, Nanjing, Jiangsu 210000, China; 3 Maitao Zhou, Department of Anesthesiology, The 904th Hospital of the Joint Logistic Support Force of PLA, Wuxi, Jiangsu 214000, China)

**Keywords:** Esketamine, Fascia iliac compartment block, Postoperative analgesia, Ropivacaine, Total hip arthroplasty

## Abstract

**Objective:**

To study the efficacy of esketamine as an adjuvant for fascia iliaca compartment block (FICB) on postoperative analgesia in elderly patients undergoing total hip arthroplasty(THA).

**Methodology:**

This study is a single-center prospective randomized controlled study. Sixty elderly patients who underwent THA at the 904th Hospital of the Joint Logistics Support Force of PLA from August 2025 to December 2025 were randomly divided into the observation group and the control group by the random number table method. The observation group conducted FICB with esketamine at concentration of 0.3 mg/kg combined with 30 mL of 0.375% ropivacaine, while the control group conducted FICB with 30 mL of 0.375% ropivacaine alone. Postoperative pain intensity, intraoperative anesthetic consumption, first press time of patient-controlled intravenous analgesia (PCIA) pump , number of effective PCIA pump presses, frequency of rescue analgesia, duration of sensory block, patient satisfaction and adverse reactions were compared between two groups.

**Results:**

The observation group showed a significantly lower VAS score both at rest and exercise T6(P<0.05), T12(P<0.05).Whether at rest or at exercise, comparison of the VAS score trends over time between two groups showed statistically significant differences(P<0.05). Intraoperative anesthetic consumption, number of effective PCIA pump presses and frequency of rescue analgesia within 48 hours after opreation were also significantly lower in the observation group (P < 0.05). Duration of sensory block and time for PCIA pump first press were significantly longer in the observation group (P < 0.05).

**Conclusion:**

Esketamine used as an adjuvant in FICB is safe and effective for elderly patient undergoing THA, providing effective pain relief, prolonging postoperative analgesia duration, with few adverse reactions and high patient satisfaction.

***Registration Number:*** ChiCTR2500107235

## INTRODUCTION

Currently, number of elderly patients with end-stage hip joint diseases such as hip fractures and femoral head necrosis is increasing. Total hip arthroplasty(THA) has become an important treatment for such diseases.^1^ However, the severe postoperative pain of THA is not conducive to early and rapid recovery after operation.^2^ In addition, most elderly patients often suffer from various underlying diseases and have low surgical tolerance, which leads to adverse reactions after opreation. Therefore, it is important to select appropriate anesthetic drug and anesthetic regimen for THA and postoperative recovery.

Relevant studies had applied FICB to THA in elderly patient with good results.^3^,^4^ However, the block time and analgesic effect were easily affected by simple safe dose of local anesthetics. Therefore, adjuvant is often used to enhance the block effect.^5^ Some studies showed that esketamine was used as an adjuvant for nerve block to provide better postoperative analgesia and widely used in brachial plexus, femoral nerve and other nerve block.^6^,^7^ This paper studies the efficacy of esketamine as an adjuvant for FICB on postoperative analgesia in elderly patient undergoing THA, which is beneficial for optimizing the clinical anesthesia scheme of THA.

## METHODOLOGY

This is a single-center prospective randomized controlled study. Sixty elderly patients undergoing THA at the 904th Hospital of the Joint Logistics Support Force of PLA between August 2025 to December 2025 were selected as study subjects. Researchers who did not participate in the implementation or data collection of this study used computer-generated random number tables to divide 60 patients into the observation group and the control group(n=30). The allocation results were sealed in opaque envelopes. After patients who met the inclusion criteria were admitted, anesthesia nurse who did not participate in this study retrieved the corresponding envelope in the order of patient admission and prepared the corresponding anesthesia block drugs before operation based on the allocation information in the envelope. The personnel responsible for postoperative pain assessment and data collection were also blinded to the patients’ group assignments.

### Sample size calculation:

Based on the pilot study results of VAS score of (1.70±0.84) and (2.43±0.77) for the observation group and control group respectively at 6 hours after operation, withα=0.05 and 1-β=0.90, the sample size was calculated as 27 by PASS 15.0. Considering the dropout rate of 10%, the final sample size was determined as 30 per group.

### Ethical Approval:

This study was approved by the Ethics Committee of the 904th Hospital of the Joint Logistics Support Force of PLA (Approval No. [2025] 0702 ; date : July 10, 2025). The informed consent was signed by the patient, and the registration was completed at the Chinese Clinical Trial Registry in August 2025 (Registration number : ChiCTR2500107235).

### Inclusion criteria:

- No limitation to gender, age 65-80 years old.
- American Society of Anesthesiologists (ASA): II-III.
- Body Mass Index (BMI) : 18-25 kg / m^2^.
- Elective unilateral the THA.
- Complete clinical data.

### Exclusion criteria:

- Contraindications to using esketamine.
- Undergoing multi-site opreation.
- Peripheral nervous system diseases involving the lower limbs.
- Operative duration was ≥ 2 hours.
- Estimated intraoperative blood loss was ≥ 600 mL.

The observation group was aged between 65 and 80, with an average age of (70.19 ± 3.15); BMI: 18.8~24.7kg/m^2^, with an average of (21.90±1.68) kg/m^2^; Gender ratio: 21 males and 9 females; ASA classification: 12 cases at level II and 18 cases at level III. The control group was aged 65-79, with an average age of (69.34±3.21); BMI: 18.3-24.8kg/m^2^, with an average of (21.97±2.34) kg/m^2^; Gender ratio: 22 males and 8 females; ASA classification: 13 cases at level II and 17 cases at level III. There was no statistically significant difference in age, BMI, gender ratio, and ASA classification between two groups (P>0.05).

### Anesthesia process:

### (1) Preoperative nerve block:

Patients were routinely fasting from food and water before operation. After entering the operating room, peripheral intravenous access was established, vital signs were routinely monitored, and invasive arterial blood pressure monitoring was performed. Ultrasound-guided FICB was performed and the specific steps were as follows. Firstly, the ultrasonic probe was placed on the side of the anterior superior iliac spine near the line between the navel and the anterior superior iliac spine. After the iliac muscle and the anterior superior iliac spine were identified, the lateral end of the probe was adjusted to rotate medially 15°. Then, 30 ml of 0.375% ropivacaine hydrochloride(control group) or 30 ml of 0.375 % ropivacaine hydrochloride with esketamine at dose of 0.3 mg / kg(observation group) was injected into that site, when the ultrasound showed that the needle tip was placed below the deep iliac circumflex artery. After injection, the deep iliac circumflex artery was moved up and the iliac muscle was moved down. Finally, the block plane was measured after 10 minutes.

### (2)Induction and maintenance of anesthesia:

Intravenous injection of midazolam(0.05mg/kg), sufentanil (0.5μg/kg), rocuronium(0.6mg/kg) and propofol(1.5mg/kg) was conducted according to patient’s condition. After completing the intravenous induction, tracheal intubation was performed and the anesthesia machine was connected for mechanical ventilation. Total intravenous anesthesia was used throughout the operation. Continuous intravenous infusion of remifentanil at the rate of 0.1-0.3 μg/(kg·min) and propofol at the rate of 4-6mg/(kg·h) was maintained with intermittent boluses of rocuronium (0.2-0.3mg/kg) added as needed during the procedure. The bispectral index (BIS) was maintained between 40 and 60 throughout the operation.

### (3)Postoperative analgesia:

Patient-controlled intravenous analgesia (PCIA) was used in all patients after operation. The analgesic drug consisted of butorphanol tartrate (8 mg) and tropisetron (5 mg) and diluted with 0.9% sodium chloride solution to 150 ml. The pump was set with a background infusion rate of 2 ml/h, a patient-controlled bolus dose of 2 ml and a lockout interval of 15 minutes. When the VAS score of patient was greater than five postoperatively, intramuscular butorphanol(1 mg) was administered with a minimum interval of four hours between radjacent injections.

### Main outcome measures:

Postoperative analgesic effect was evaluated by visual analogue scale ( VAS ) score. The VAS score of patient at 6 h ( T6 ), 12 h ( T12 ), 24 h ( T24 ) and 48 h ( T48 ) after operation were recorded at rest. The VAS score range was 0-10.The higher the score, the more severe the pain.^8^

### Secondary outcome measures:

VAS score at exercise at each postoperative time point; the consumption of propofol and remifentanil during operation; duration of sensory block; first press time of PCIA pump within 48 hours after operation; number of effective PCIA pump presses; frequency of rescue analgesia. Satisfaction with analgesic efficacy (0 indicated very dissatisfied, 10 indicated very satisfied) and the occurrence of adverse reactions such as nausea and vomiting, drowsiness, bradycardia, hypotension and delirium within 48 hours after operation.

### Statistical analysis:

Statistical analysis was performed using SPSS 25.0. Continuous data was expressed as mean ± standard deviation (SD), and categorical data was expressed as frequencies (percentages). Categorical data was compared using the chi-square test. Independent sample t-test was used for comparing continuous data between two groups. Moreover, Repeated measures analysis of variance(ANOVA) was used to assess the VAS score within-group differences over time, while the independent sample t-test was applied for between-group comparisons at each time interval. The continuous data had been checked and met the conditions for conducting the independent sample t-test. P-value < 0.05 was considered statistically significant.

## RESULTS

As shown in Table-I, the observation group showed a significantly lower VAS score both at rest and exercise T6(at rest: t=-6.530, P<0.001; at exercise: t=-6.630, P<0.001), T12(at rest: t=-8.098, P<0.001; at exercise: t=-4.193, P<0.001). Whether at rest or exercise, comparison of the VAS score trends over time between two groups showed statistically significant differences (at rest: F=27.771, P<0.001; at exercise: F=16.097, P<0.001).

**Table-I:** Comparison of VAS scores at rest and exercise at different postoperative time point between the two groups(*x̅*±*s*, n=30)

VAS score at different time points	Observation group	Control group	t/F	P
VAS score at rest(T6)	1.03±0.67	2.20±0.71	-6.530	<0.001
VAS score at rest(T12)	2.10±0.48	3.13±0.51	-8.098	<0.001
VAS score at rest(T24)	2.27±0.74	2.47±0.63	-1.128	0.264
VAS score at rest(T48)	2.00±0.53	2.10±0.61	-0.682	0.498
Within group difference for VAS score at rest			27.771	<0.001
VAS score at exercise(T6)	1.83±0.79	3.20±0.81	-6.630	<0.001
VAS score at exercise(T12)	3.02±0.96	4.07±0.94	-4.193	<0.001
VAS score at exercise(T24)	2.90±0.88	3.23±0.77	-1.553	0.126
VAS score at exercise(T48)	2.74±0.65	2.98±0.74	-1.490	0.142
Within group difference for VAS score at rest			16.097	<0.001

As shown in Table-II, there were significant differences in the consumption of propofol and remifentanil, the duration of sensory block, the first press time of PCIA pump, the number of effective PCIA pump presses, frequency of rescue analgesia within 48h after operation and Satisfaction with analgesic efficacy between two groups (P < 0.05). As shown in Table-III, There was no significant difference in adverse reactions between two groups (p>0.05).

**Table-II:** Comparison of propofol and remifentanil consumption, duration of sensory block, the first press time of PCIA pump, number of effective PCIA pump presses, frequency of rescue analgesia within 48h postoperatively and Satisfaction with analgesic efficacy(*x̅*±*s*, n=30).

Group	Consumption of anesthesia drugs during operation		Usage of PCIA pump within 48 hours after operation			Duration of sensory block / min	Satisfaction with analgesic efficacy
	Propofol /mg	Remifentanil /μg	The first press time/h	Number of effective presses	Frequency of rescue analgesia		
Observation group	146.17±41.67	750.85±90.07	12.38±0.93	0.70±0.47	0.30±0.47	731.63±66.27	9.30±0.47
Control group	180.43±40.05	838.71±98.04	10.87±0.87	1.37±0.85	0.83±0.59	620.30±59.73	8.67±0.61
*t*	-3.248	-3.615	6.457	-3.766	-3.877	6.834	4.535
*P*	0.002	0.001	<0.001	<0.001	<0.001	<0.001	<0.001

**Table-III:** Comparison of adverse reactions between the two groups(%, n=30)

Group	Incidence of adverse reactions/n(%)					
	Nausea and vomiting/n(%)	Drowsiness /n(%)	Bradycardia /n(%)	Hypotension /n(%)	Delirium /n(%)	Total incidence of adverse reactions/n(%)
Observation group	2(6.67)	2(6.67)	1(3.33)	1(3.33)	0(0.00)	6(20.00)
Control group	2(6.67)	1(3.33)	2(6.67)	3(10.00)	0(0.00)	8(26.67)
*χ^2^*	0	0.351	0.351	1.071	0	0.373
*P*	1	0.554	0.554	0.301	1	0.524

## DISCUSSION

Ropivacaine is a commonly used local anesthetic for FICB, and 0.375% concentration of ropivacaine is effective and safe for elderly THA patient.^9^ As the dextrorotatory isomer of ketamine, esketamine has milder neurological adverse reactions and potential neuroprotective effect^10^,^11^, with a low risk of neurotoxicity at low doses, making it suitable as a nerve block adjuvant.^12^ Related studies have found that the use of 0.3 mg/kg dose of esketamine as an adjuvant combined with ropivacaine in THA for elderly patient was safe and effective.^13^ In this study, 0.3 mg/kg dose of esketamine was selected as the adjuvant dose in combination with 0.375% ropivacaine for FICB.

This study indicates that the anesthesia regimen used in the observation group has a better postoperative analgesic effect, longer analgesic duration and significant advantages in early postoperative analgesia, which can be attributed to the following mechanisms. As an NMDA receptor antagonist, esketamine inhibits the binding of glutamate to NMDA receptors, thereby suppressing neuronal hyperexcitability and reducing pain signal transmission. Meanwhile, the combination of esketamine and ropivacaine for FICB achieved effective complementarity of different analgesic mechanisms, accelerated the onset time, and produced a synergistic multimodal analgesic effect.^14^ Some researches also suggest that esketamine could reduce the release of inflammatory factors, thereby providing better analgesic effect.^15^ The main metabolite of esketamine is 6-hydroxydemethylamine, which can achieve analgesic effect by regulating pain transmission pathways, neurotransmitter release and inhibiting inflammatory reactions.^16^ Clinical studies have confirmed that esketamine used as an adjuvant for peripheral nerve block has a good analgesic effect and can effectively prolong the duration of nerve block analgesia^13^, which is consistent with the conclusion of this study.

This study results also shows that the observation group used significantly less anesthesia drugs during operation.^17^ The combination of ropivacaine and esketamine improves the quality and safety of anesthesia block, thereby reducing the consumption of propofol and remifentanil, which is consistent with the conclusion in reference.^18^ The increase in patient satisfaction without an increase in the incidence of adverse events such as drowsiness and delirium in this study should be related to the high affinity of esketamine for NMDA receptors, which can reduce dose related adverse reactions.^19^,^20^ Related studies used esketamine for postoperative analgesia in patients undergoing gastrointestinal examinations and traumatic fractures, without producing adverse events such as hypotension. The incidence of postoperative nausea and vomiting has significantly decreased.^21^

### Limitations:

The single-center, small-sample design may lead to the potential selection bias. Moreover, the postoperative follow-up is scheduled 48 hours after discharge, making it difficult to evaluate the effect of esketamine on chronic postsurgical pain and its long-term safety.

## CONCLUSION

The application of esketamine as an adjuvant for FICB in elderly patients undergoing THA can enhance postoperative analgesia to a certain extent, prolong the duration of analgesia, improve patient satisfaction, and low incidence of adverse reactions.

### Recommendations:

Further research will be conducted on the use of multi center, large sample size, and different doses of ketamine as an adjuvant to enhance the persuasiveness of the research results.
